# Integration of multi-imaging technique and immunohistochemistry for the development of a predictive nomogram for axillary lymph node metastasis in breast cancer

**DOI:** 10.3389/fendo.2025.1659639

**Published:** 2025-12-04

**Authors:** Jinxiao Chen, Mengyao Zhao, Zeyang Dong, Pengzhao Zhang

**Affiliations:** 1 Department of Ultrasound, Lishui Hospital of Wenzhou Medical University, The First Affiliated Hospital of Lishui University, Lishui People’s Hospital, Lishui, Zhejiang, China; 2 The Second School of Clinical Medicine, Zhejiang Chinese Medical University, Hangzhou, Zhejiang, China; 3 Department of Catheterization Laboratory, Lishui Hospital of Wenzhou Medical University, The First Affiliated Hospital of Lishui University, Lishui People’s Hospital, Lishui, Zhejiang, China

**Keywords:** breast cancer, axillary lymph node metastasis, ultrasound, magnetic resonance imaging, immunohistochemistry, line drawing

## Abstract

**Background:**

Axillary lymph node metastasis (ALNM) significantly influences staging, treatment, and prognosis in breast cancer. Current assessment relies on invasive procedures such as sentinel lymph node biopsy (SLNB) and axillary lymph node dissection (ALND), which carry substantial morbidity. A reliable, noninvasive method for preoperative ALNM prediction is urgently needed.

**Methods:**

This retrospective study included 528 female breast cancer patients without distant metastasis treated at Lishui People’s Hospital between February 2019 and February 2024. Preoperative ultrasound (US) and magnetic resonance imaging (MR) features of primary tumors and axillary lymph nodes, along with immunohistochemistry (IHC) markers, were analyzed. Univariate and multivariate logistic regression analyses were performed to identify independent predictors of ALNM, and a nomogram was constructed. Model performance was assessed using receiver operating characteristic (ROC) analysis, calibration curves, decision curve analysis (DCA), and five-fold cross-validation.

**Results:**

Multivariate analysis identified six independent predictors of ALNM: SWE min (≤7.16 kPa), microcalcification, suspicious lymph nodes on US and MR, ADC value (≤0.955 × 10^−^³ mm²/s), and HER-2 positivity (all P < 0.05). The nomogram incorporating these variables achieved area under the ROC curve (AUC) values of 0.854 in the training cohort and 0.826 in the validation cohort. Calibration curves showed good agreement between predicted and observed probabilities. DCA demonstrated favorable net clinical benefit, and five-fold cross-validation confirmed the model’s stability with a mean AUC of 0.812.

**Conclusions:**

A nomogram integrating multimodal imaging features and IHC markers provides a noninvasive, accurate tool for preoperative prediction of ALNM in breast cancer. This model may assist in individualized surgical planning and reduce unnecessary axillary interventions. Further validation in multicenter prospective studies is warranted.

## 1 Introduction

According to the 2020 global cancer statistics, breast cancer has become the most commonly diagnosed malignancy worldwide. In China, both the incidence and mortality of breast cancer are increasing rapidly, with a growth rate approximately twice the global average, ranking among the highest globally (1, 2). Breast cancer cells exhibit a strong propensity for hematogenous and lymphatic dissemination, with axillary lymph node metastasis (ALNM) being the earliest and most common route of spread. ALNM status is closely associated with clinical staging, treatment strategies, and patient prognosis (3).Currently, ALNM is primarily assessed through sentinel lymph node biopsy (SLNB) or axillary lymph node dissection (ALND). While both approaches provide histopathological confirmation, they are invasive and carry a risk of complications such as lymphedema, nerve injury, and sensory deficits, which significantly affect patients’ quality of life (4, 5). Moreover, the false-negative rate of SLNB is highly dependent on the operator’s level of experience (6). These limitations underscore the urgent need for a noninvasive, accurate preoperative method to predict ALNM, facilitating individualized and precision-based surgical management of breast cancer.

Current imaging modalities for assessing ALNM include ultrasound (US), magnetic resonance imaging (MR), mammography (MG), computed tomography (CT), and positron emission tomography-computed tomography (PET-CT). PET-CT is limited in clinical use due to its high radiation exposure and cost. MG is highly sensitive to calcifications but lacks sufficient spatial resolution to accurately evaluate axillary lymph node morphology and vascularity, resulting in low sensitivity. US enables real-time assessment of lymph node size, cortical thickness, vascularity, and elasticity, but metastatic nodes often lack specific features in the early stage, increasing the risk of underdiagnosis. MR offers excellent soft-tissue contrast and provides a detailed view of the tumor and surrounding structures; however, its diagnostic utility is constrained by coil size, motion artifacts, and overlap in imaging features between benign and malignant nodes. Thus, reliance solely on imaging features for ALNM detection remains insufficient.

ALNM in breast cancer is a complex, multistep process influenced by tumor growth and cellular proliferation. The morphology of the primary tumor correlates with its biological characteristics and undergoes changes during lymph node metastasis (7, 8). Previous studies have demonstrated a significant association between the imaging features of primary breast tumors and ALNM (9, 10). Furthermore, immunohistochemical (IHC) markers such as estrogen receptor (ER), human epidermal growth factor receptor 2 (HER-2), and progesterone receptor (PR) contribute to pathological changes and are linked to ALNM occurrence (11–13). However, most prior research has been limited to clinical-pathological data or single imaging modalities, with few studies integrating multi-modality imaging of both primary tumors and axillary lymph nodes to predict ALNM.

This study integrated US and MR imaging features of primary breast tumors and axillary lymph nodes with IHC markers to identify independent risk factors for ALNM using univariate and multivariate analyses. A nomogram prediction model was developed and validated to estimate the probability of ALNM. This intuitive and practical tool enables preoperative risk assessment of ALNM, supporting clinical decision-making and individualized treatment planning.

## 2 Materials and methods

### 2.1 Participants

This retrospective analysis was conducted on 528 female breast cancer patients without distant metastasis treated at Lishui People’s Hospital, Zhejiang Province, from February 2019 to February 2024. All patients underwent primary tumor resection combined with ipsilateral axillary lymph node dissection or SLNB. Based on postoperative ALNM status, patients were classified into positive and negative groups and randomly divided into a training set (n=369) and a validation set (n=159) at a 7:3 ratio. This study was approved by the institutional ethics committee, with informed consent waived. Inclusion criteria were as follows: 1) female patients; 2) newly diagnosed with a single breast cancer lesion; 3) availability of clear and complete preoperative MR and US imaging along with comprehensive clinical data; 4) preoperative 14G core needle biopsy of the primary tumor with completed IHC analysis; and 5) underwent tumor resection with axillary lymph node dissection or SLNB, with postoperative pathological confirmation of breast cancer. The exclusion criteria were as follows: 1) inflammatory breast cancer; 2) prior biopsy of the tumor before imaging, potentially affecting image interpretation; 3) pregnancy or lactation; 4) presence of distant metastases or other malignancies; 5) history of radiotherapy, chemotherapy, or endocrine therapy; and 6) incomplete imaging or clinical data.

### 2.2 Imaging examination

#### 2.2.1 Equipment and materials

Breast MR imaging was performed using a 1.5T Siemens Aera scanner equipped with an 8-channel dedicated breast phased-array coil. US examinations were conducted using a Mindray Resona 7 system with a linear-array transducer operating at 7.0–14.0 MHz.

#### 2.2.2 Methods and observation indicators

US and MR images were interpreted according to the 2013 American College of Radiology BI-RADS classification. All images were retrieved from the hospital’s PACS system. Two radiologists with over 10 and 15 years of experience in breast imaging independently reviewed the images, and discrepancies were resolved through consensus with a senior radiologist.

Breast MR imaging was performed using a 1.5T scanner with patients in the prone position and both breasts naturally suspended within the dedicated coil. The scanning range included the entire breasts and bilateral axillary regions. Sequences acquired included diffusion-weighted imaging (DWI), fat-suppressed T2-weighted imaging, and dynamic contrast-enhanced T1-weighted imaging. Gadolinium-based contrast (gadopentetate dimeglumine, 0.1 mmol/kg) was administered via the dorsal hand vein at 2.0 mL/s, followed by a 15 mL saline flush. On DWI images, regions of interest (ROIs) were manually delineated within the solid tumor area, avoiding necrotic, hemorrhagic, or cystic regions. The apparent diffusion coefficient (ADC) was measured three times and averaged. Time-signal intensity curves (TICs) were generated using FUNCTOOL software. MR features evaluated included breast composition, tumor size, shape, margin, internal enhancement pattern, TIC type, T1 and fat-suppressed T2 signal intensity, DWI signal characteristics, ADC values, and axillary lymph node status. Lymph nodes were considered metastatic if they exhibited any of the following: significant enlargement, round shape, irregular margins, cortical thickening, or absent hilum (14). These criteria are in accordance with established radiological guidelines for the evaluation of axillary lymph nodes in breast cancer. Lymph nodes larger than 1 cm in short-axis diameter were considered enlarged and suspected to be metastatic. However, histopathological examination remains the gold standard for confirming ALNM.

US examination was performed using a Mindray Resona 7 system equipped with a high-frequency linear-array transducer (L14-5, 7.0–14.0 MHz) in breast mode, with image parameters optimized based on individual patient characteristics. Patients were positioned supine with arms raised overhead to fully expose the breasts and axillae. Bilateral breasts and lymphatic drainage areas were scanned clockwise starting from the nipple. Suspected lesions were evaluated in multiple planes, and the longest diameter was recorded. Color Doppler imaging was applied first, followed by strain elastography (SE) and shear wave elastography (SWE). To ensure data quality, the subsequent elastography examinations adhered to a standardized acquisition and quality control protocol, as detailed in section 2.2.3 below. US features evaluated included lesion size, orientation, shape, margin, internal echogenicity, posterior acoustic features, calcification type, vascularity (graded 0–III based on Adler’s semi-quantitative classification) (15), qualitative SE scoring (16), and SWE-based quantitative stiffness measurements (17). Axillary lymph nodes were considered metastatic if any of the following were observed: rounded or lobulated shape, indistinct margins, focal or diffuse cortical thickening, longitudinal-to-transverse ratio >1, or loss of hilum structure (14).

The image parameters were optimized based on each patient’s specific characteristics, following a standardized approach to ensure consistency within each patient’s examination.

#### 2.2.3 Elastography acquisition protocol and quality control

##### 2.2.3.1 SE

Gentle, rhythmic free-hand compressions were applied perpendicular to the skin surface. The compression amplitude was controlled to maintain the on-screen deformation index between 3 and 4.

The elastogram was interpreted in real-time. The elastographic patterns were qualitatively graded from ‘soft’ to ‘hard’ based on the degree of deformation of the lesion relative to the surrounding normal glandular tissue. For semi-quantitative analysis, the strain ratio (lesion-to-fat ratio) was calculated by placing one ROI within the stiffest part of the lesion and a second ROI of equal size on the adjacent subcutaneous fat tissue at the same depth.

##### 2.2.3.2 SWE

The transducer was held stable with minimal pre-compression. Patients were asked to briefly hold their breath. The SWE color map was monitored until a stable and homogeneous fill was achieved within the region of interest (ROI), typically within 2–5 seconds. Examinations with significant, persistent void areas within the lesion were discarded and repeated.

A 2-mm diameter circular Q-Box was manually placed on the stiffest area (typically red on the color scale) within the lesion to obtain the maximum elasticity (SWEmax). Three additional Q-Boxes were then sequentially placed on the stiffest, softest, and most heterogeneous areas to record the mean (SWEmean), minimum (SWEmin), and standard deviation (SWEsd) values, respectively.

**Figure 1:**
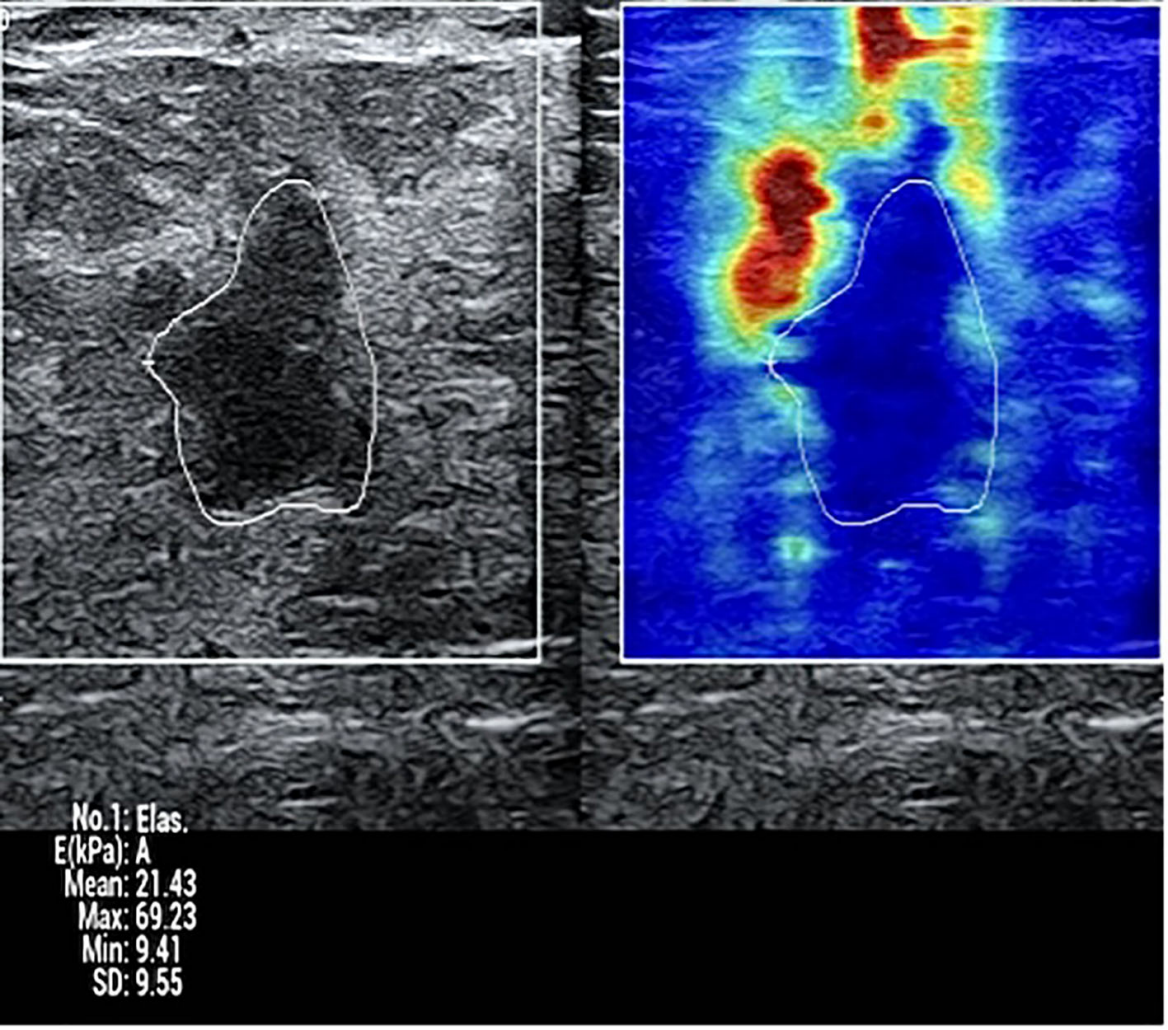
Representative SWE image of a breast lesion.

The entire acquisition and measurement process was repeated three times for each lesion. The median value from these three independent measurements was used for final analysis to ensure robustness (**Figure 1**).

#### 2.2.3.3 Inter- and intra-observer agreement

To evaluate the reproducibility of SWE measurements, an agreement study was conducted on a random subset of 50 patients. For intra-observer agreement, one radiologist repeated measurements on the same images with a two-week interval. For inter-observer agreement, a second blinded radiologist performed independent measurements. The consistency of SWE parameters was assessed using the intraclass correlation coefficient (ICC), with values >0.75 indicating good reliability.

### 2.3 Axillary lymph node metastasis

During surgery, sentinel lymph nodes were identified using a combination of indocyanine green and methylene blue dyes and excised for biopsy. Patients with positive sentinel nodes underwent axillary lymph node dissection. Resected lymph nodes were processed into paraffin-embedded sections and examined microscopically by senior pathologists to determine the presence of metastasis.

### 2.4 Immunohistochemistry interpretation criteria

IHC staining was performed using a fully automated Roche platform to evaluate the expression of ER, PR, HER-2, Ki-67, CK5/6, and P63 in breast cancer tissue samples. ER and PR were considered positive if >1% of tumor cell nuclei showed staining. HER-2 expression was scored as 0, 1+, 2+, or 3+: scores of 0 and 1+ were defined as negative, 3+ as positive, and 2+ required further evaluation using fluorescence *in situ* hybridization (FISH); FISH-positive cases were classified as HER-2 positive. Ki-67 expression was assessed based on the proportion of nuclei with brown granules, with >20% defined as high expression and ≤20% as low expression, in accordance with the 2024 Chinese Anti-Cancer Association guidelines (18). CK5/6 positivity was defined as cytoplasmic staining in ≥5% of tumor cells, and P63 positivity as nuclear staining in >10% of tumor cells.

### 2.5 Statistical analysis

Statistical analyses were performed using SPSS 22.0 and R software (version 3.6.1). Continuous variables with normal distribution were expressed as mean ± standard deviation ( $\bar{x}$ ± s), while non-normally distributed data were presented as median (interquartile range). Categorical variables were summarized as counts and percentages. Differences in categorical data between groups were assessed using the χ² test or Fisher’s exact test. Statistically significance was set at P<0.05. Variables significant in univariate analysis within the training set were included in multivariate logistic regression to identify independent risk factors for ALNM, based on which a nomogram was developed. The model’s performance was evaluated in both training and validation cohorts using the Hosmer-Lemeshow goodness-of-fit test, receiver operating characteristic (ROC) curve, and clinical decision curve analysis to assess calibration, discrimination, and clinical utility. Five-fold cross-validation was applied to assess model stability. Points were assigned to each independent factor category to calculate a total score, which corresponds to the predicted probability of preoperative ALNM (**Table 1**).

**Table 1:** Statistical assignment table.

Group	Indicators	0	1	2	3	4
	LN metastasis	None	Present			
X1	US max diameter		≦2cm	>2cm		
X2	Orientation		Horizontal position	Vertical position		
X3	Shape		Regular	Irregular		
X4	Margin		Well-defined	Ill-defined	Spiculated	
X5	Echo		Isoechoic/Hyperechoic	Mixed echogenicity/ Hypoechoic		
X6	Posterior echo		Unchanged/Enhancement	Attenuation/Mixed		
X7	Calcification type	None	Macrocalcification	Microcalcification		
X8	Alder blood flow grading		Grade 0/I	Grade II/III		
X9	SWE(mean)					
X10	SWE(max)					
X11	SWE(min)					
X12	SWE(SD)					
X13	SE		≦3	>3		
X14	Strain Ratio					
X15	Suspicious LN on US	None	Present			
X16	MR max diameter		≦2cm	>2cm		
X17	MR glandular type		Fatty	Scattered fibroglandular	Heterogeneously dense	Extremely dense
X18	Internal enhancement	None	Homogeneous	Heterogeneous	Rim	Septation
X19	TIC type		Persistent	Plateau	Washout	
X20	T1 signal		Hypointense	Isointense	Hyperintense	Heterogeneous
X21	fsT2 signal		Hypointense	Isointense	Hyperintense	Heterogeneous
X22	DWI signal		Bright	Not bright		
X23	ADC value					
X24	Suspicious LN on MR	None	Present			
X25	Lesion location		Left	Right		
X26	Quadrant		UOQ	Non-UOQ		
X27	Age		≤40	40-60	≥60	
X28	Ki-67		<20%	≥20%		
X29	ER	Negative	Positive			
X30	PR	Negative	Positive			
X31	HER⁃2	Negative	Positive			
X32	CK5/6	Negative	Positive			
X33	P63	Negative	Positive			
X34	Molecular subtype	Luminal A	Luminal B(HER⁃2+)	Luminal B(HER⁃2-)	HER⁃2 overexpression	Triple-negative(TNBC)

LN, Lymph node; US, Ultrasound; MR, Magnetic resonance; SWE, Shear wave elastography; Max, Maximum; Min, Minimum; SD, Standard deviation; SE, Strain elastography; TIC, Time-signal intensity curve; DWI, Diffusion-weighted imaging; ADC, Apparent diffusion coefficient; UOQ, Upper outer quadrant; ER, Estrogen receptor; PR, Progesterone receptor; HER-2, Human epidermal growth factor receptor; TNBC, Triple-negative breast cancer.

### 2.6 Ethics statement

The study was approved by the ethics board of Lishui People’s Hospital (no. 2024-012-13) and individual consent for this retrospective analysis was waived. All procedures conformed to the Declaration of Helsinki. All methods were carried out in accordance with relevant guidelines and regulations.

## 3 Results

### 3.1 Baseline clinical and pathological characteristics

A total of 528 female breast cancer patients were included in this study, with ages ranging from 16 to 89 years (mean ± SD: 54.3 ± 13.1 years). The most common pathological type was invasive ductal carcinoma (n = 394), followed by ductal carcinoma *in situ* (n = 76), invasive lobular carcinoma (n = 12), papillary carcinoma (n = 34), mucinous carcinoma (n = 7), lobular carcinoma *in situ* (n = 2), and medullary carcinoma (n = 3). Among all patients, 387 had no ALNM, and 141 were ALNM-positive. In the ALNM-positive group, invasive ductal carcinoma was the predominant subtype (121 cases, 85.21%), followed by ductal carcinoma *in situ* (17 cases, 11.97%).

A total of 528 breast cancer patients were randomly assigned to a training cohort (n = 369) and a validation cohort (n = 159) in a 7:3 ratio. No significant differences were observed between the two cohorts in terms of imaging features and clinicopathological variables (P > 0.05), indicating good comparability.

### 3.2 Univariate analysis of ALNM predictors

Univariate analysis was performed on US, MR imaging features, and clinicopathological variables from 369 breast cancer patients in the training cohort. Detailed results are presented in **Table 2**.

**Table 2:** Univariate analysis of imaging features and clinicopathologic indicators in breast cancer patients with and without ALNM.

Indicators		LN metastasis (n%)		*Z/χ^2^*	*P*
		None(271)	Present(98)		
Age groups	≤40	34(12.55)	18(18.37)	2.459	0.292
	40-60	117(43.17)	43(43.88)
	≥60	120(44.28)	37(37.75)
Lesion location	Left	144(53.14)	52(53.06)	0.000	0.990
	Right	127(46.86)	46(46.94)
Quadrant	UOQ	107(39.48)	53(54.08)	6.245	0.012
	Non-UOQ	164(60.52)	45(45.92)
Ki-67	<20%	97(35.79)	21(21.42)	6.828	0.009
	≥20%	174(64.21)	77(78.58)
ER	Negative	76(28.04)	28(28.57)	0.010	0.921
	Positive	195(71.96)	70(71.43)
PR	Negative	96(35.42)	35(35.71)	0.003	0.959
	Positive	175(64.58)	63(64.29)
HER⁃2	Negative	213(78.60)	49(50.00)	28.59	<0.001
	Positive	58(21.40)	49(50.00)
CK5/6	Negative	212(78.23)	70(71.43)	1.847	0.174
	Positive	59(21.77)	28(28.57)
P63	Negative	233(85.98)	92(93.88)	4.277	0.039
	Positive	38(14.02)	6(6.12)
Molecular subtype	Luminal A	84(31.00)	13(13.27)	0.890	0.306
	Luminal B( HER⁃2+)	92(33.95)	37(37.76)
	Luminal B( HER⁃2-)	32(11.81)	15(15.31)
	HER⁃2 overexpression	23(8.49)	21(21.43)
	Triple-negative(TNBC)	40(14.76)	12(12.24)
US max diameter group	≤2cm	116(42.80)	28(28.57)	6.127	0.013
	>2cm	155(57.20)	70(71.43)		
Orientation	Vertical position	9(3.32)	5(5.10)	0.233	0.630
	Horizontal position	262(96.68)	93(94.90)		
Shape	Regular	34(12.55)	8(8.16)	1.371	0.242
	Irregular	237(87.45)	90(91.84)		
Margin	Well-defined	19(7.01)	3(3.06)	5.413	0.067
	Ill-defined	194(71.59)	64(65.31)		
	Spiculated	58(21.40)	31(31.63)		
Echo	Homogeneous	11(4.06)	3(3.06)	0.018	0.893
	Heterogeneous	260(95.94)	95(96.94)		
Posterior echo	No attenuation	170(62.73)	52(53.06)	2.808	0.094
	Attenuation	101(37.27)	46(46.94)		
Calcification type	None	129(47.60)	21(21.43)	20.875	<0.001
	Macrocalcification	6(2.21)	2(2.04)		
	Microcalcification	136(50.19)	75(76.53)		
Blood flow	None/Minimal	96(35.42)	31(31.63)	0.458	0.498
	Abundant	175(64.58)	67(68.37)		
SE	≤3	179(66.05)	57(58.16)	1.943	0.163
	>3	92(33.95)	41(41.84)		
SWE mean		23.92(16.95,33.22)	23.08(18.16,31.6)	-0.029	0.977
SWE max		90.29(56.14,138.7)	98.45(59.36,143.7)	-1.003	0.316
SWE min		6.62(4.18,10.31)	5.57(3.63,7.08)	-2.927	0.003
SWE SD		12.72(7.9,18.05)	12.29(7.93,19.16)	-0.218	0.828
Suspicious LN on US	None	230(84.87)	13(13.27)	164.111	<0.001
	Present	41(15.13)	85(86.73)
MR glandular type	Fatty	15(5.54)	2(2.04)	3.561	0.264
	Scattered fibroglandular	135(49.81)	64(65.31)		
	Heterogeneously dense	112(41.33)	31(31.63)		
	Extremely dense	9(3.32)	1(0.10)		
MR max diameter group	≤2cm	115(42.44)	29(29.59)	4.989	0.026
	>2cm	156(57.56)	69(70.41)		
Shape	Regular	65(23.99)	9(9.18)	9.835	0.002
	Irregular	206(76.01)	89(90.82)		
Margin	Well-defined	45(16.61)	7(7.14)	10.655	0.005
	Ill-defined	189(69.74)	66(67.35)		
	Spiculated	37(13.65)	25(25.51)		
Internal enhancement	Homogeneous	45(16.61)	16(16.33)	1.392	0.846
	Heterogeneous	80(29.52)	31(31.63)		
	Rim	112(41.33)	36(36.73)		
	Septation	34(12.18)	15(15.31)		
TIC type	Persistent	37(13.65)	4(4.08)	10.097	0.006
	Plateau	56(20.66)	14(14.29)		
	Washout	178(65.69)	80(81.63)		
T1	Hypointense	0(0.00)	0(0.00)	1.232	0.540
	Isointense	253(93.36)	94(95.92)		
	Hyperintense	10(3.69)	3(3.06)		
	Heterogeneous	8(2.95)	1(1.02)		
fsT2	Hypointense	16(5.90)	5(5.10)	0.330	0.954
	Isointense	64(23.62)	24(24.49)		
	Hyperintense	106(39.11)	36(36.73)		
	Heterogeneous	85(31.37)	33(33.67)		
DWI	Bright	137(50.55)	51(52.04)	0.064	0.801
	Not bright	134(49.45)	47(47.96)		
ADC value		0.86(0.76,1.00)	0.79(0.70,0.90)	-4.066	<0.001
Suspicious LN on MR	None	259(95.57)	28(28.57)	186.931	<0.001
	Present	12(4.43)	70(71.43)

LN, Lymph node; US, Ultrasound; MR, Magnetic resonance; SWE, Shear wave elastography; Max, Maximum; Min, Minimum; SD, Standard deviation; SE, Strain elastography; TIC, Time-signal intensity curve; DWI, Diffusion-weighted imaging; ADC, Apparent diffusion coefficient; UOQ, Upper outer quadrant; ER, Estrogen receptor; PR, Progesterone receptor; HER-2, Human epidermal growth factor receptor; TNBC, Triple-negative breast cancer.

#### 3.2.1 Association of US and SWE features with ALNM

Univariate analysis of US features in the training cohort, including 98 ALNM-positive and 271 ALNM-negative cases, revealed significant differences in tumor maximum diameter, calcification morphology, SWEmin, and suspicious lymph nodes on US (P < 0.05). The ALNM-positive group exhibited a higher proportion of tumors larger than 2 cm, increased microcalcifications, and more frequent Adler blood flow grades II and III, along with lower SWEmin values; the optimal SWEmin cutoff was ≤7.16 kPa as determined by the Youden index.

#### 3.2.2 Association of MRI parameters with ALNM

MR parameters, including maximum diameter, tumor shape, margin, TIC, ADC, and suspicious lymph nodes, also differed significantly between groups (P < 0.05). The ALNM-positive tumors more commonly displayed irregular shapes and margins, washout-type TIC patterns, and lower ADC values, with the optimal ADC cutoff determined as ≤0.955×10^−^³ mm²/s.

#### 3.2.3 Association of clinicopathological and immunohistochemical features with ALNM

Clinicopathological factors, including tumor quadrant, Ki-67, HER-2, and P63 expression, were significantly different (P < 0.05). ALNM-positive cases were more frequently located in the upper outer quadrant, showed higher Ki-67 expression and P63 negativity, whereas HER-2 negativity was more prevalent in the ALNM-negative group.

### 3.3 Identification of independent predictors by multivariable logistic regression

Variables with significant differences in univariate analysis, including US and MR imaging features and clinicopathological parameters, were incorporated into a multivariate logistic regression model. The analysis identified SWEmin, calcification morphology, suspicious lymph nodes on US, ADC value, suspicious lymph nodes on MR, and HER-2 expression as independent risk factors for ALNM in breast cancer patients (P < 0.05), while other factors showed no significant association (P > 0.05) (**Table 3**).

**Table 3:** Multivariate logistic regression analysis to predict ALNM.

Characteristics	β	*S.E*	*Z*	*P*	*OR*	*95% C.I.*	
						Lower limit	Upper limit
Shape	0.695	0.825	0.843	0.399	2.004	0.398	10.099
MR max diameter	1.218	1.518	0.802	0.423	3.379	0.172	66.273
Margin							
Well-defined					1.0 (Reference)		
Ill-defined	0.414	1.014	0.409	0.683	1.513	0.207	11.037
Spiculated	1.559	1.111	1.403	0.161	4.755	0.539	41.966
TIC							
Persistent					1.0 (Reference)		
Plateau	-0.449	1.158	-0.387	0.698	0.639	0.066	6.176
Washout	0.561	1.021	0.550	0.583	1.753	0.237	12.967
Suspicious LN on MR	3.895	0.728	5.351	<.001	49.174	11.807	204.805
ADC value	-0.072	0.018	-4.021	<.001	0.930	0.898	0.964
US max diameter group	-1.220	1.523	-0.801	0.423	0.295	0.015	5.842
Calcification type							
No- Calcification					1.0 (Reference)		
Macrocalcification	1.298	1.099	1.181	0.238	3.661	0.425	31.542
Microcalcification	2.345	0.617	3.803	<.001	10.431	3.115	34.931
Suspicious LN on US	3.123	0.584	5.348	<.001	22.712	7.231	71.339
SWE min	-0.211	0.072	-2.916	0.004	0.810	0.703	0.933
Quadrant	-0.082	0.503	-0.163	0.871	0.921	0.344	2.468
ki67	0.790	0.565	1.397	0.162	2.203	0.727	6.673
Her2	1.070	0.518	2.064	0.039	2.916	1.056	8.054
P63	-1.263	0.804	-1.572	0.116	0.283	0.059	1.366
constant	-0.357	1.932	-0.185	0.853	0.699	0.016	30.825

ALNM, Axillary lymph node metastasis; LN, Lymph node; US, Ultrasound; MR, Magnetic resonance; SWE, Shear wave elastography; Max, Maximum; Min, Minimum; TIC, Time-signal intensity curve; ADC, Apparent diffusion coefficient; HER-2, Human epidermal growth factor receptor.

### 3.4 Development, performance, and validation of the predictive nomogram

**Figure 2:**
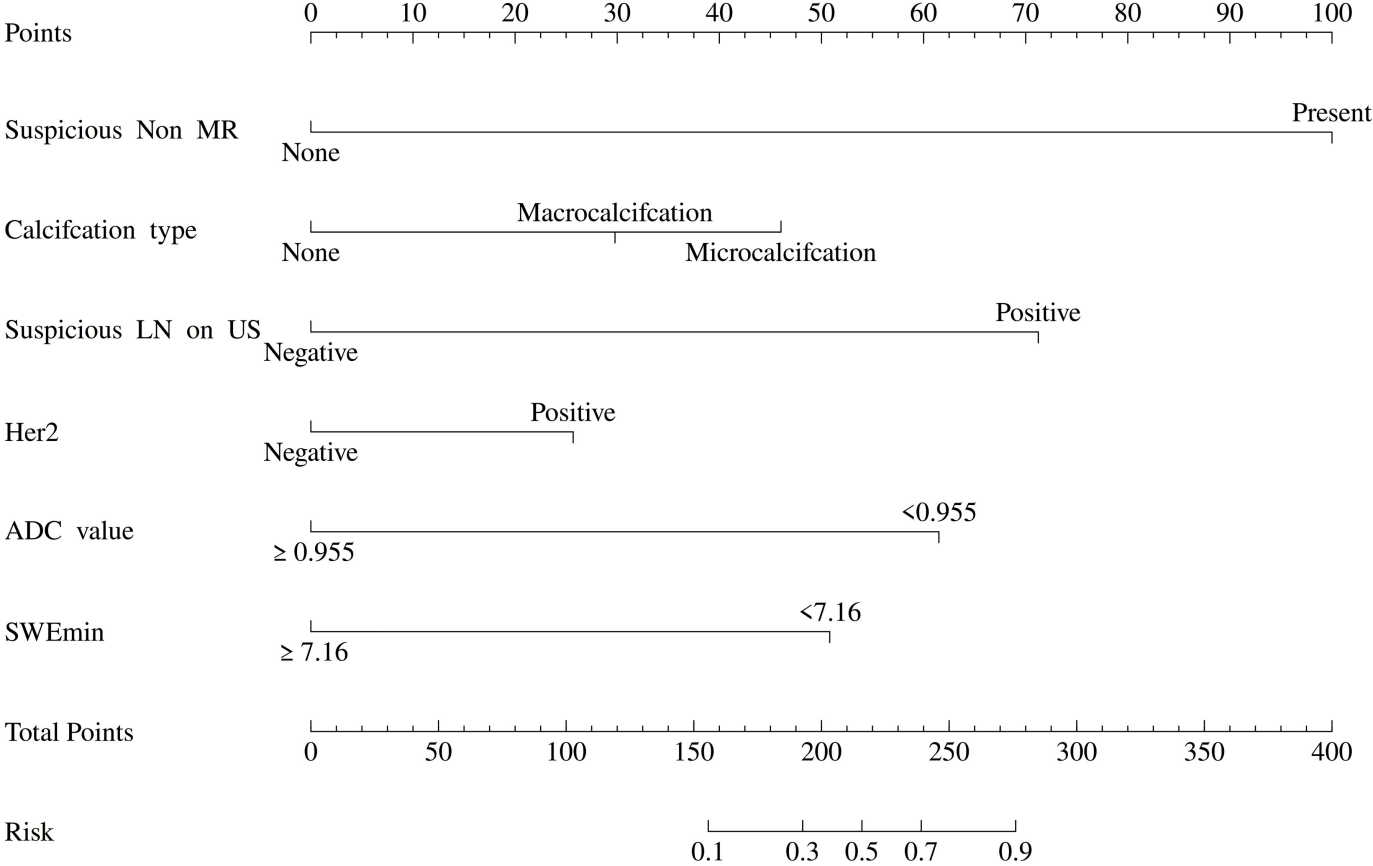
Risk prediction nomogram for ALNM in breast cancer patients.

A nomogram was constructed based on the results of multivariate logistic regression, assigning points to each predictor and calculating a total score. Higher total scores corresponded to an increased probability of ALNM (**Figure 2**).

The nomogram model, developed using US and MR imaging features combined with IHC markers from the training cohort, was validated in the independent validation cohort. Model performance was assessed using the ROC curve. Calibration and goodness-of-fit were evaluated via the Hosmer–Lemeshow test and calibration curve. Clinical utility was assessed using decision curve analysis, and model stability was evaluated through 5-fold cross-validation.

**Figure 3:**
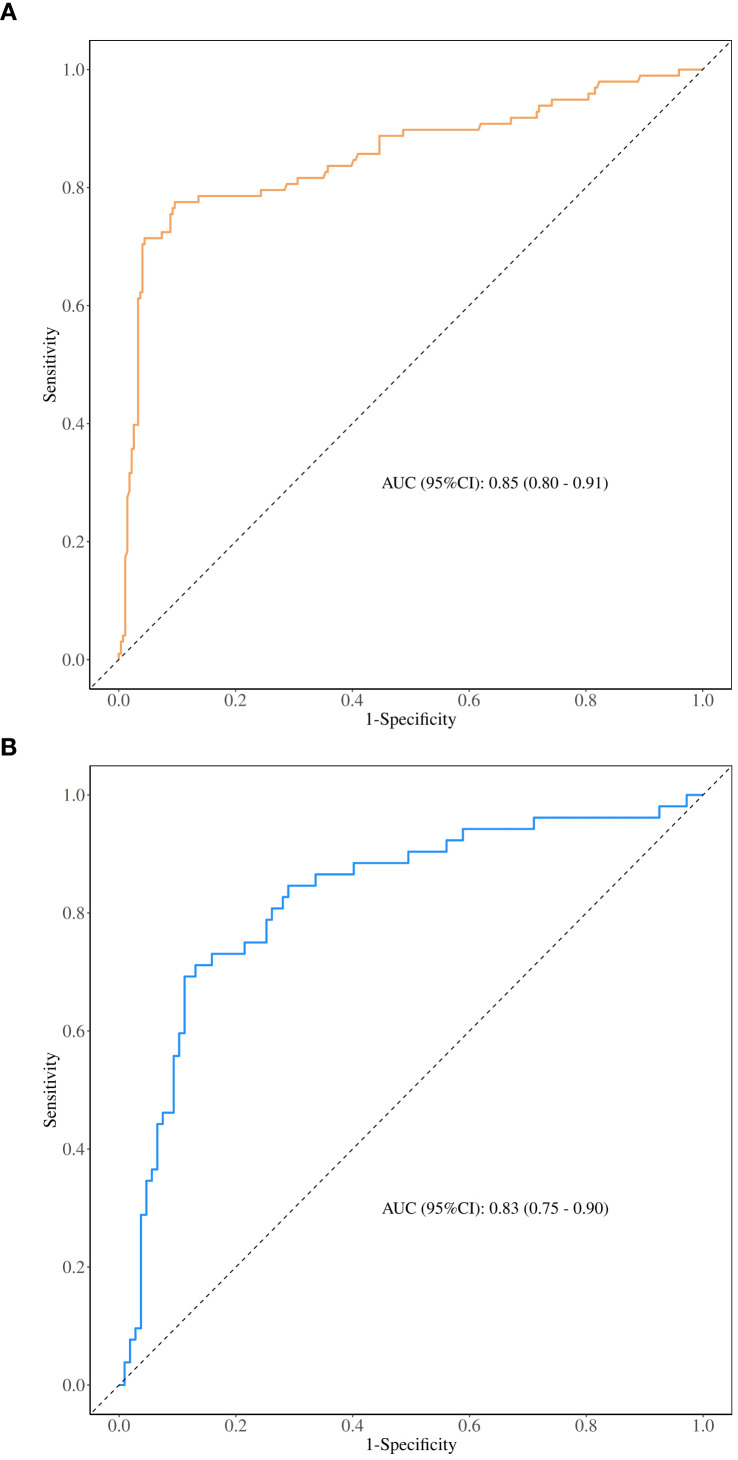
**(A)** Training set ROC curve of the nomogram **(B)** Validation set ROC curve of the nomogram.

The nomogram demonstrated good diagnostic performance in both the training and validation cohorts, with AUCs of 0.854 (95% CI: 0.803–0.906) and 0.826 (95% CI: 0.753–0.898), respectively (**Table 4**) (**Figures 3A, B**). Calibration curves and Hosmer-Lemeshow tests confirmed model fit.

**Table 4:** Comparison of diagnostic efficacy of the training set as well as the validation set of the axillary lymph node metastasis columnar mapping model for breast cancer.

	AUC(95% CI)	Accuracy	Sensitivity	Specificity	Positive predictive value	Negative predictive value
Training set	0.854 (0.803-0.906)	0.870	0.776	0.904	0.918	0.745
Validation set	0.826 (0.753-0.898)	0.805	0.712	0.850	0.858	0.698

AUC, Area under the curve.

**Figure 4:**
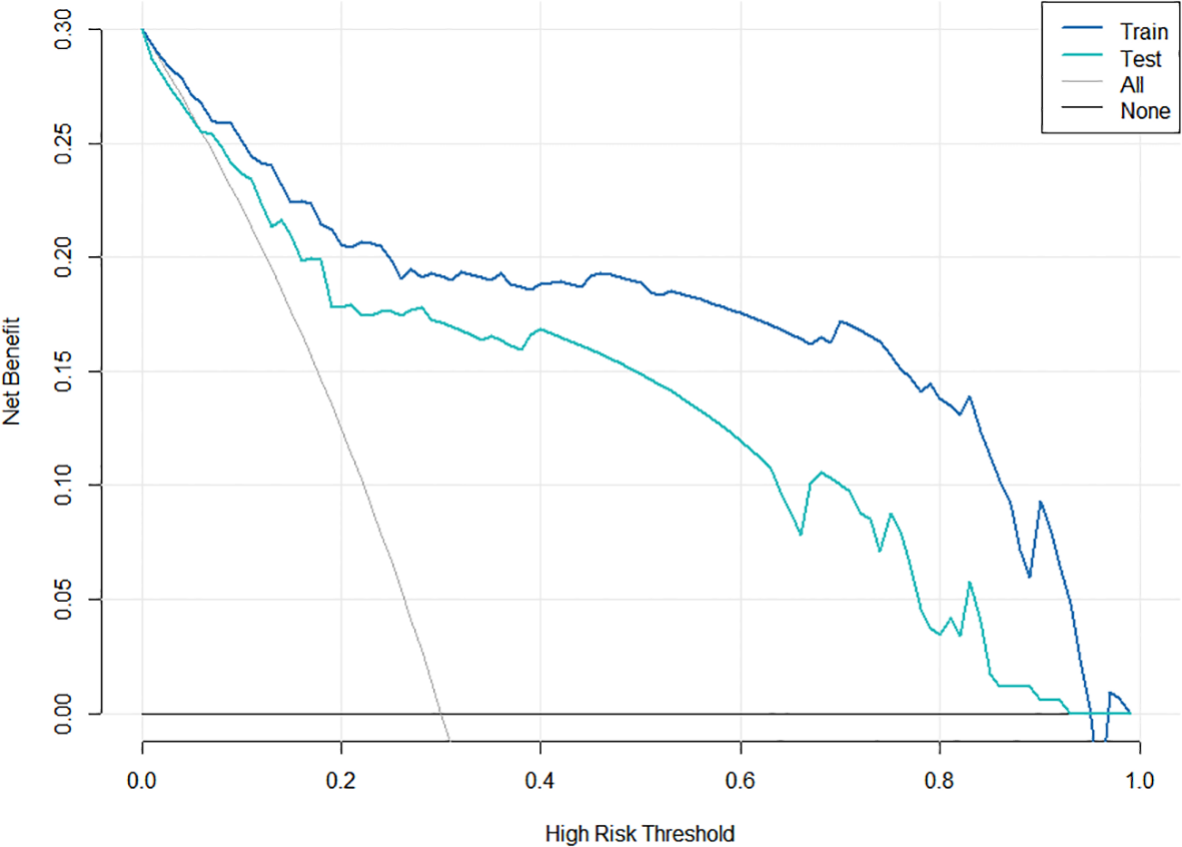
Clinical decision curve of the nomogram model for predicting ALNM in breast cancer.

Clinical decision curve analysis was used to quantify the net benefit across various threshold probabilities, where the y-axis represents net clinical benefit and the x-axis represents the threshold probability. The gray and dashed lines correspond to the assumptions that all patients are ALNM-positive and ALNM-negative, respectively. In the training cohort, a threshold range of 0.10–0.99 yielded a maximum net benefit of 0.528, while in the validation cohort a threshold range of 0.10–0.99 yielded a maximum net benefit of 0.496. These results indicate that the nomogram model has good clinical utility (**Figure 4**). Five-fold cross-validation demonstrated model stability (mean AUC 0.812).

## 4 Discussion

Breast cancer has surpassed lung cancer as the most prevalent malignancy worldwide, with both incidence and mortality rates in China continuing to rise (19). Despite advances in early detection, distant metastasis and tumor recurrence remain the primary causes of death. ALNM is the most common route of dissemination and plays a crucial role in staging and prognostication (20, 21).

This study explored the predictive value of multimodal imaging features combined with IHC markers for ALNM in breast cancer and constructed a nomogram model to assist clinical decision-making. While imaging techniques such as ultrasound and MRI, along with IHC markers, play significant roles in the preoperative assessment of ALNM, their accuracy has yet to reach the level of histopathological evaluation. SLNB and ALND are widely regarded as the gold standard for assessing ALNM. SLNB, which involves pathological examination of the sentinel node, is effective in identifying metastatic lymph nodes and offers lower complication risks compared to ALND, such as lymphedema and nerve injury (22, 23). Moreover, evidence suggests that ALND may not provide additional survival benefit for patients with only sentinel node metastases but contributes to overtreatment and unnecessary morbidity (22, 24, 25). Therefore, a reliable, noninvasive method for preoperative ALNM assessment is urgently needed.

Tumor size showed a significant association with ALNM in univariate analysis but was not an independent predictor in multivariate regression, possibly due to inter-operator measurement variability.

Notably, microcalcification emerged as an independent predictor (OR = 10.431, P < 0.001) and was included in the final model. Microcalcifications are considered a surrogate for necrosis and aggressive tumor behavior. SWE also showed promise, as tumors in the ALNM group exhibited significantly lower SWEmin values—likely reflecting altered stromal architecture or desmoplastic response—while other SWE parameters (SWEmean, SWEmax), though previously suggested, were not predictive in our cohort, possibly due to equipment differences or sample size limitations (26).

In addition, US-detected suspicious lymph nodes with cortical thickening, round shape, and loss of hilar architecture were significantly associated with ALNM, confirming the diagnostic value of nodal morphology on US (27).

Although tumor morphology, size, margin, and TIC types were statistically significant in univariate analysis, none proved to be independent predictors in multivariate models. This may be attributed to selection bias or limited sample size. Importantly, ADC values were inversely associated with ALNM, with higher ADC values serving as protective factors (OR = 0.93). The best cutoff identified was 0.955 × 10^−^³ mm²/s. These findings are consistent with reports linking lower ADC values to high tumor cellularity and restricted diffusion (28, 29).

MRI-detected suspicious lymph nodes, characterized by enlarged size, cortical thickening, irregular borders, or absence of fatty hilum, also independently predicted ALNM, reaffirming their clinical utility in preoperative evaluation (27).

Among molecular biomarkers, HER-2 overexpression was identified as an independent risk factor for ALNM (OR = 2.916, P < 0.05), consistent with its role in promoting cell proliferation and invasion (11). Although Ki-67 and P63 showed significant differences in univariate analysis, neither entered the final model. The inconsistency in Ki-67 may stem from heterogeneous cutoff definitions. P63’s dual role in different subtypes and the existence of multiple isoforms might explain its variable association with metastasis (30).

A nomogram incorporating six independent variables (SWEmin, microcalcification, suspicious lymph nodes on US and MR, ADC value, and HER-2 status) was developed. The model demonstrated excellent discriminatory ability with AUCs of 0.854 and 0.826 in the training and validation cohorts, respectively. Calibration curves showed strong agreement between predicted and actual probabilities, and decision curve analysis indicated substantial clinical utility. Five-fold cross-validation further confirmed the model’s robustness.

In this study, we developed a non-invasive predictive model for preoperative ALNM by integrating multimodal imaging features with IHC markers. This approach, combining imaging and molecular biomarkers, offers a more comprehensive and individualized method for assessing ALNM risk, which has not been widely explored in previous literature.

Several studies have explored the role of imaging techniques alone in predicting ALNM. Zhao et al. demonstrated the utility of MRI in identifying ALNM, showing that MRI features such as tumor size, shape, and margin can effectively predict axillary lymph node involvement (31). However, their model focused on MRI alone, without integrating IHC markers like HER-2 or Ki-67, which could improve predictive accuracy. Similarly, Yilmaz et al. found that ultrasound elastography could serve as a valuable tool for ALNM detection (32). While ultrasound elastography is a useful technique for assessing tissue stiffness and detecting abnormalities, it also lacks the ability to provide detailed molecular information that might refine risk stratification.

Our study builds upon these findings by integrating both ultrasound and MRI features with IHC markers, such as HER-2 expression. The inclusion of molecular biomarkers allows for a more nuanced prediction of ALNM risk, adding a layer of biological insight to imaging-based assessments. This combination of multimodal imaging and molecular markers is an important innovation in the current literature, as it offers a more comprehensive, non-invasive alternative to the traditional SLNB and ALND, which are invasive and carry potential risks such as lymphedema and nerve injury.

Furthermore, the incorporation of HER-2 expression in our model is particularly significant, as it has been shown to correlate with aggressive tumor behavior and higher rates of metastasis. Our model offers a more precise and individualized approach to preoperative ALNM prediction, potentially reducing unnecessary invasive procedures and improving clinical decision-making. While previous studies have explored the individual use of ultrasound or MRI in ALNM prediction, few have combined these imaging modalities with molecular biomarkers such as HER-2 expression in a single predictive framework (33). Compared to the HRCT-based approach, our model combining ultrasound, MRI, and IHC markers demonstrated higher predictive accuracy and performed more precisely in preoperative axillary lymph node metastasis prediction, particularly in early screening and surgical decision-making for low-risk patients (34).

This was a retrospective single-center study with a relatively small sample size and no external validation. Variability in image acquisition and interpretation across operators and equipment may have influenced the results. Inter-observer agreement was not evaluated. Future multicenter, prospective studies with standardized imaging protocols and quality control measures are warranted to enhance the generalizability of the model.

## 5 Conclusion

Our study confirms the feasibility and predictive value of a nomogram integrating US, MR, and IHC features for noninvasive preoperative assessment of ALNM in breast cancer. The model may help reduce unnecessary axillary interventions and optimize individualized treatment strategies. Further validation in larger, multicenter cohorts is needed to support its clinical application.

## Data Availability

The raw data supporting the conclusions of this article will be made available by the authors, without undue reservation.
